# The genome sequence of the Kite-tailed Robberfly, *Machimus atricapillus* (Fallén, 1814)

**DOI:** 10.12688/wellcomeopenres.19080.1

**Published:** 2023-03-08

**Authors:** Sam Thomas, Ryan Mitchell, Liam M. Crowley, Natural History Museum Genome Acquisition Lab, University of Oxford and Wytham Woods Genome Acquisition Lab, Darwin Tree of Life Barcoding collective, Wellcome Sanger Institute Tree of Life programme, Wellcome Sanger Institute Scientific Operations: DNA Pipelines collective, Tree of Life Core Informatics collective, Darwin Tree of Life Consortium

**Affiliations:** 1 Natural History Museum, London, UK; 2 University of Oxford, Oxford, UK

**Keywords:** Machimus atricapillus, Kite-tailed Robberfly, genome sequence, chromosomal, Diptera

## Abstract

We present a genome assembly from an individual male *Machimus atricapillus* (the Kite-tailed Robberfly; Arthropoda; Insecta; Diptera; Asilidae). The genome sequence is 268.6 megabases in span. Most of the assembly is scaffolded into six chromosomal pseudomolecules, including the X and Y sex chromosomes. The mitochondrial genome has also been assembled and is 16.3 kilobases in length. Gene annotation of this assembly on Ensembl identified 10,978 protein coding genes.

## Species taxonomy

Eukaryota; Metazoa; Ecdysozoa; Arthropoda; Hexapoda; Insecta; Pterygota; Neoptera; Endopterygota; Diptera; Brachycera; Muscomorpha; Asiloidea; Asilidae; Asilinae; *Machimus*; *Machimus atricapillus* (Fallén, 1814) (NCBI:txid2794001).

## Background

*Machimus atricapillus* (Fallén, 1814) is a medium-sized, grey-brown robberfly (Asilidae) with a body length of 11 to 18 mm ( Stubbs & Drake, 2014; van den Broek & Schulten, 2017; Wolff *et al.,* 2018). It is one of a group of several similar *Machimus* (Loew, 1849) species. Males can be easily separated from all other UK Asilidae by the presence of a projection on the hind edge of sternite eight which is clothed with black hairs and normally indented to form a shape similar to the tail of a kite ( *Milvus* Lacépède, 1799) ( Stubbs & Drake, 2014), giving it the common name of Kite-tailed Robberfly. Females are more difficult to identify but can be distinguished from similar UK *Machimus* species based on a combination of features including leg colour and the hairing of the sternites ( Smart, 2005).

Larvae of other *Machimus* species are known to be soil dwelling and feed on the larvae of beetles (Coleoptera) in the families Scarabaeidae, Chrysomelidae, and Curculionidae ( Musso, 1983). Adults are predaceous on a range of insects, with most prey consisting of other Diptera ( Parmenter, 1942). In the UK the adult flight period spans from May to October with a peak in July and August ( Stubbs & Drake, 2014). Adults are often found sunning themselves on vantage points such as fence posts and tree trunks or foliage or sitting on bare ground ( Stubbs & Drake, 2014).

*Machimus atricapillus* is widely distributed across southern Britain in open habitats with dry soils but becomes rarer in the north and has not been recorded from Ireland or most of Scotland ( Chandler, 2022; Harvey, 2018; Stubbs & Drake, 2014). *M. atricapillus* is widespread in Europe though absent from the far north ( Lehr, 1988) and is found widely though Russia as far east as Sakhalin Island ( Astakhov *et al.*, 2019) and in Iran ( Ghahari *et al.*, 2014).

*Machimus atricapillus* is treated as *Tolmerus atricapillus* (Fallén, 1814) by Lehr (1988) and most subsequent authorities, e.g. ( Astakhov *et al.*, 2019; Ghahari *et al.*, 2014; Kahanpää *et al.*, 2014; Larsen & Meier, 2004). However, this generic placement has not been accepted by UK authorities with both Stubbs and Drake (2014) and the UK Diptera checklist ( Chandler, 2022) retaining the species in *Machimus*.

The high-quality genome sequence described here is the first one reported for *M. atricapillus*, to our knowledge, and has been generated as part of the Darwin Tree of Life project. It will aid future research on the taxonomy of the genus *Machimus and the phylogeny of the wider* Asilidae as well as contributing to our understanding of the biology, physiology and ecology of *M. atricapillus*.

### Genome sequence report

**Figure 1.**
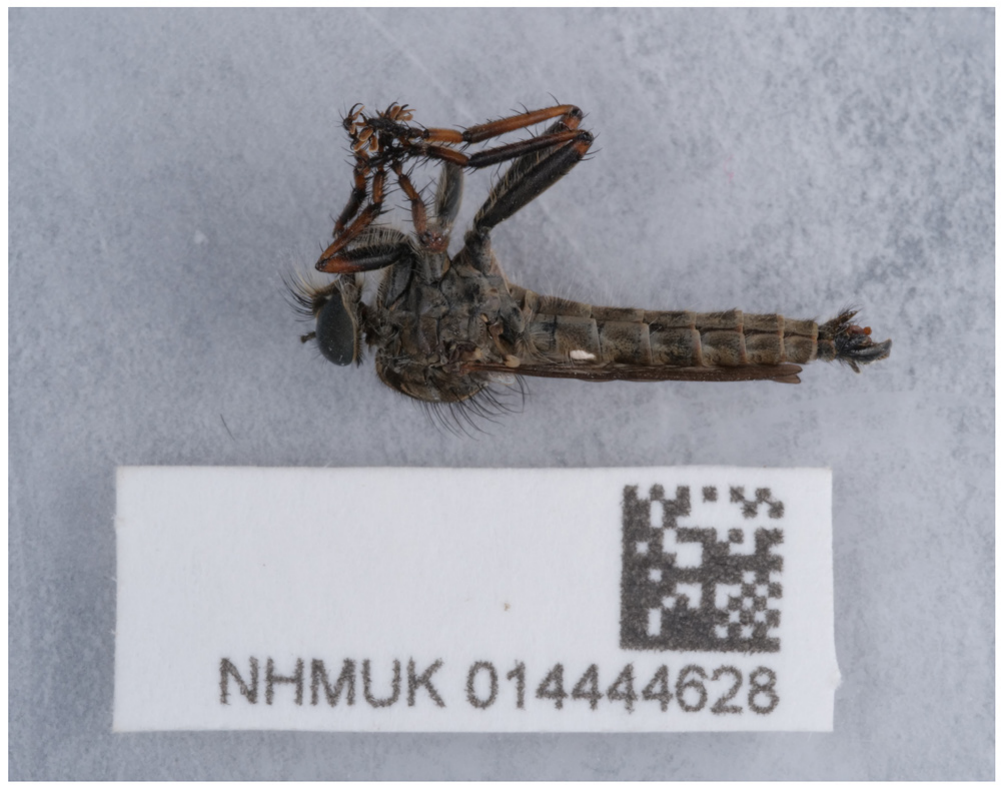
Photograph of the *Machimus atricapillus* (idMacAtri3) specimen used for genome sequencing.

The genome was sequenced from one male *Machimus atricapillus* specimen ( Figure 1) collected from Hartslock Reserve, Oxfordshire (latitude 51.511263, longitude –1.112222). A total of 47-fold coverage in Pacific Biosciences single-molecule HiFi long reads was generated. Primary assembly contigs were scaffolded with chromosome conformation Hi-C data. Manual assembly curation corrected 17 missing or mis-joins, reducing the scaffold number by 9.68%, and increasing the scaffold N50 by 5.34%.

**Figure 2.**
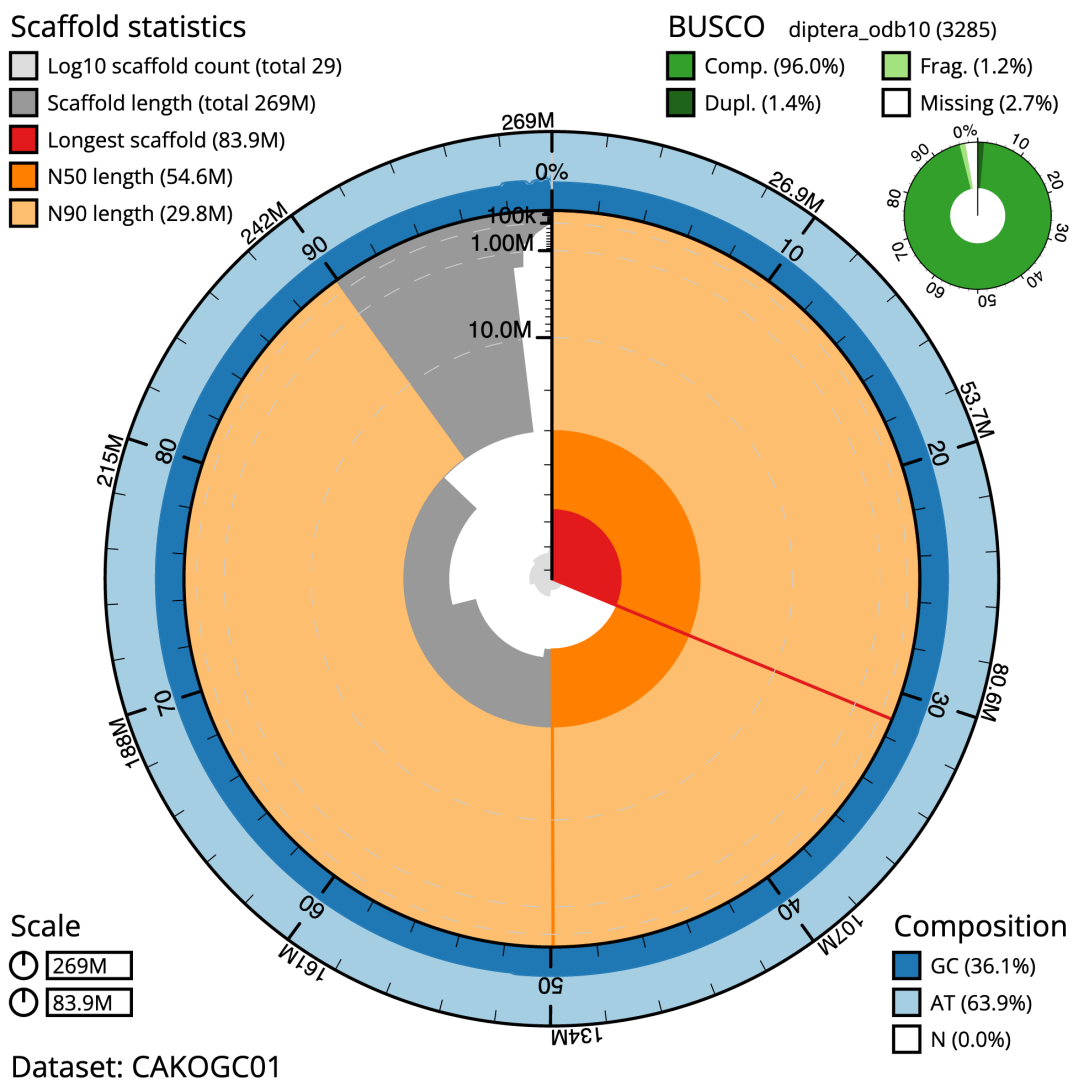
Genome assembly of *Machimus atricapillus*, idMacAtri3.1: metrics. The BlobToolKit Snailplot shows N50 metrics and BUSCO gene completeness. The main plot is divided into 1,000 size-ordered bins around the circumference with each bin representing 0.1% of the 268,644,068 bp assembly. The distribution of scaffold lengths is shown in dark grey with the plot radius scaled to the longest scaffold present in the assembly (83,901,797 bp, shown in red). Orange and pale-orange arcs show the N50 and N90 scaffold lengths (54,569,305 and 29,817,269 bp), respectively. The pale grey spiral shows the cumulative scaffold count on a log scale with white scale lines showing successive orders of magnitude. The blue and pale-blue area around the outside of the plot shows the distribution of GC, AT and N percentages in the same bins as the inner plot. A summary of complete, fragmented, duplicated and missing BUSCO genes in the diptera_odb10 set is shown in the top right. An interactive version of this figure is available at https://blobtoolkit.genomehubs.org/view/idMacAtri3.1/dataset/CAKOGC01/snail.

The final assembly has a total length of 268.6 Mb in 28 sequence scaffolds with a scaffold N50 of 54.6 Mb ( Table 1). Most (99.99%) of the assembly sequence was assigned to six chromosomal-level scaffolds, representing four autosomes and the X and Y sex chromosomes. Chromosome-scale scaffolds confirmed by the Hi-C data are named in order of size ( Figure 2– Figure 5; Table 2). The assembly has a BUSCO v5.3.2 ( Manni *et al.*, 2021) completeness of 96.0% (single 94.7%, duplicated 1.4%) using the diptera_odb10 reference set. While not fully phased, the assembly deposited is of one haplotype. Contigs corresponding to the second haplotype have also been deposited.

**Table 1.** Genome data for *Machimus atricapillus*, idMacAtri3.1.

Project accession data		
Assembly identifier	idMacAtri3.1	
Species	*Machimus atricapillus*	
Specimen	idMacAtri3	
NCBI taxonomy ID	2794001	
BioProject	PRJEB50882	
BioSample ID	SAMEA7849389	
Isolate information	idMacAtri3 idMacAtri4, female (Hi-C)	
Assembly metrics *		*Benchmark*
Consensus quality (QV)	65.1	*≥ 50*
*k*-mer completeness	100%	*≥ 95%*
BUSCO **	C:96.0%[S:94.7%,D:1.4%], F:1.2%,M:2.7%,n:3,285	*C ≥ 95%*
Percentage of assembly mapped to chromosomes	99.99%	*≥ 95%*
Sex chromosomes	X and Y chromosomes	*localised homologous pairs*
Organelles	Mitochondrial genome assembled	*complete single alleles*
Raw data accessions		
PacificBiosciences SEQUEL II	ERR8705867	
Hi-C Illumina	ERR8702790	
PolyA RNA-Seq Illumina	ERR8702790, ERR10378006	
Genome assembly		
Assembly accession	GCA_933228815.1	
*Accession of alternate haplotype*	GCA_933228755.1	
Span (Mb)	268.6	
Number of contigs	48	
Contig N50 length (Mb)	25.9	
Number of scaffolds	28	
Scaffold N50 length (Mb)	54.6	
Longest scaffold (Mb)	83.9	
Genome annotation		
Number of protein-coding genes	10,978	
Number of non-coding genes	694	
Number of gene transcripts	17,462	

* Assembly metric benchmarks are adapted from column VGP-2020 of “Table 1: Proposed standards and metrics for defining genome assembly quality” from ( Rhie *et al.*, 2021). ** BUSCO scores based on the diptera_odb10 BUSCO set using v5.3.2. C = complete [S = single copy, D = duplicated], F = fragmented, M = missing, n = number of orthologues in comparison. A full set of BUSCO scores is available at https://blobtoolkit.genomehubs.org/view/idMacAtri3.1/dataset/CAKOGC01/busco.

**Figure 3.**
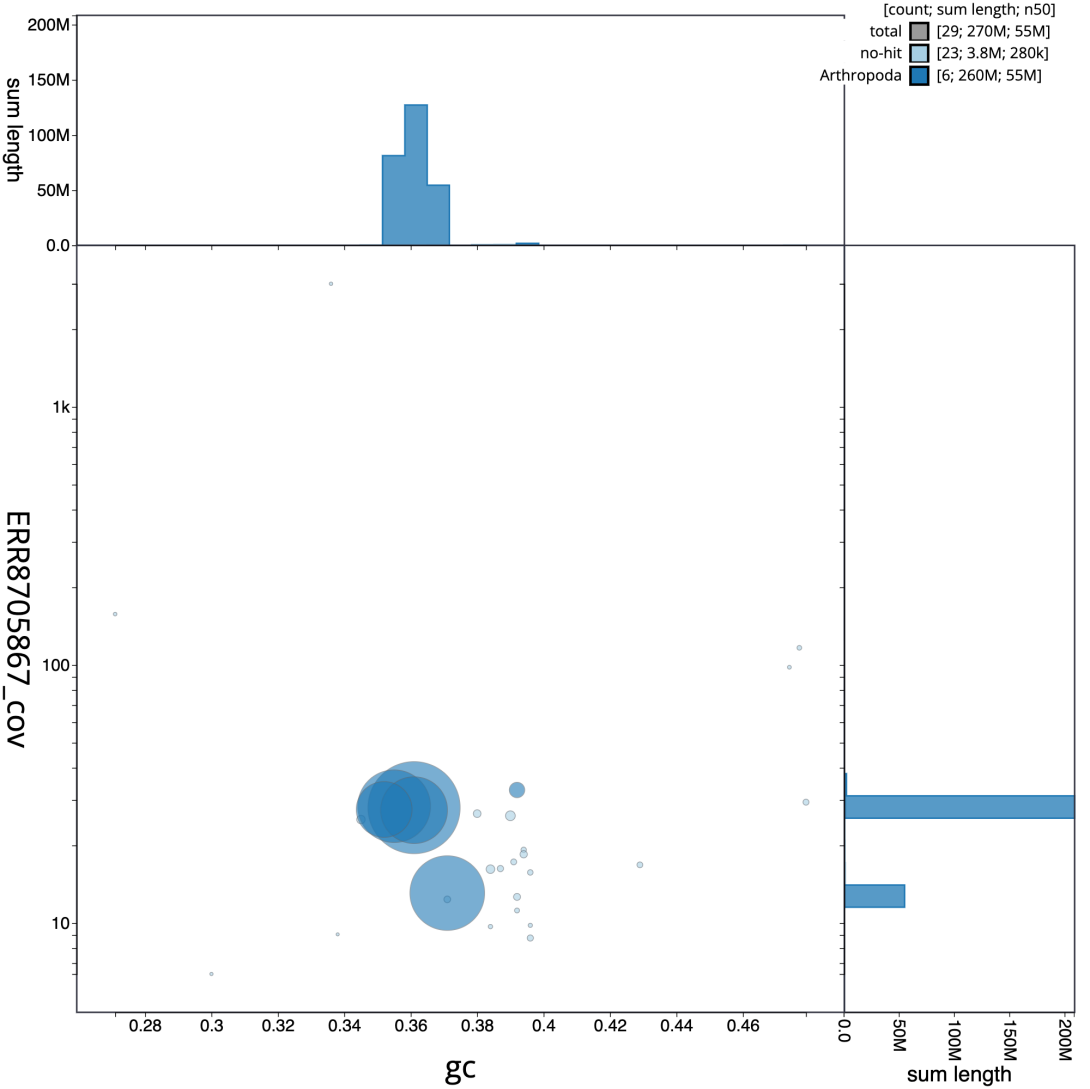
Genome assembly of *Machimus atricapillus*, idMacAtri3.1: GC coverage. BlobToolKit GC-coverage plot. Scaffolds are coloured by phylum. Circles are sized in proportion to scaffold length. Histograms show the distribution of scaffold length sum along each axis. An interactive version of this figure is available at https://blobtoolkit.genomehubs.org/view/idMacAtri3.1/dataset/CAKOGC01/blob.

**Figure 4.**
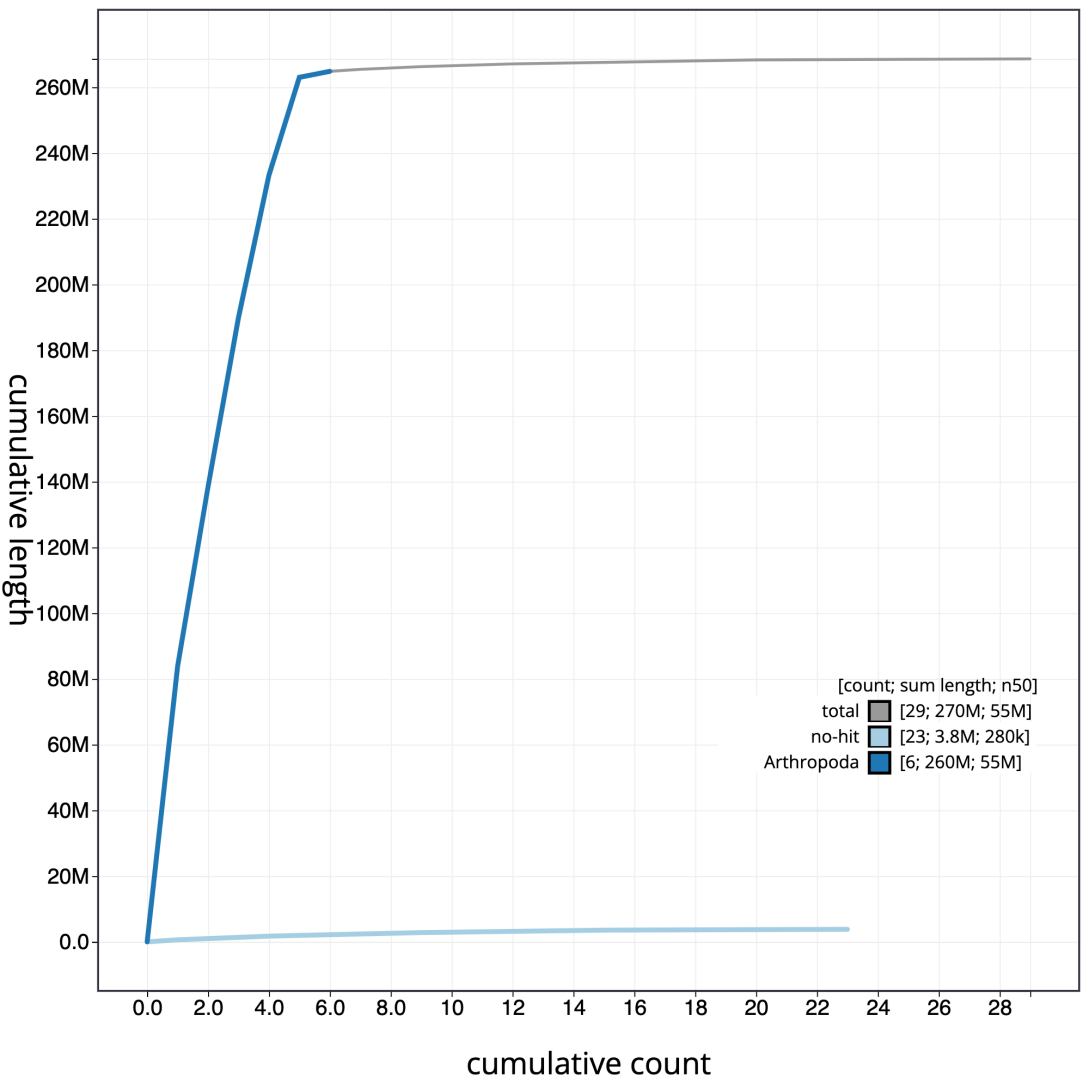
Genome assembly of *Machimus atricapillus*, idMacAtri3.1: cumulative sequence. BlobToolKit cumulative sequence plot. The grey line shows cumulative length for all scaffolds. Coloured lines show cumulative lengths of scaffolds assigned to each phylum using the buscogenes taxrule. An interactive version of this figure is available at https://blobtoolkit.genomehubs.org/view/idMacAtri3.1/dataset/CAKOGC01/cumulative.

**Figure 5.**
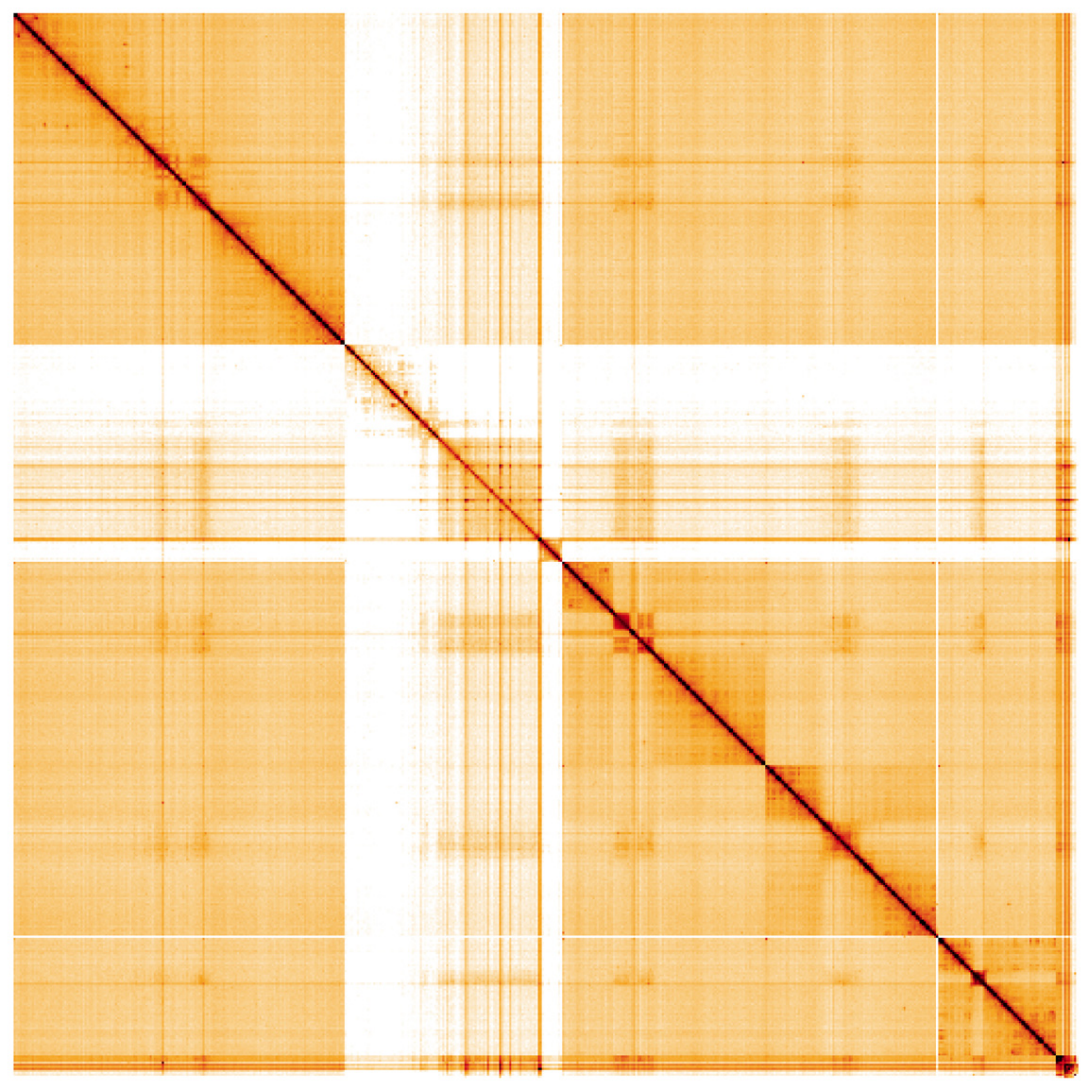
Genome assembly of *Machimus atricapillus*, idMacAtri3.1: Hi-C contact map of the idMacAtri3.1 assembly, visualised using HiGlass. Chromosomes are shown in order of size from left to right and top to bottom. An interactive version of this figure may be viewed at https://genome-note-higlass.tol.sanger.ac.uk/l/?d=fnEw__WoTp6esZWuwMdF5w.

**Table 2.** Chromosomal pseudomolecules in the genome assembly of *Machimus atricapillus*, idMacAtri3.

INSDC accession	Chromosome	Size (Mb)	GC%
OW121784.1	1	83.9	36.1
OW121786.1	2	51.48	35.5
OW121787.1	3	43.26	36.1
OW121788.1	4	29.82	35.2
OW121785.1	X	54.57	37.1
OW121789.1	Y	1.82	39.2
OW121790.1	MT	0.02	33.7
-	unplaced	3.78	39

### Genome annotation report

The *M. atricapillus* GCA_933228815.1 genome assembly was annotated using the Ensembl rapid annotation pipeline ( Table 1; https://rapid.ensembl.org/Machimus_atricapillus_GCA_933228815.1/). The resulting annotation includes 17,462 transcribed mRNAs from 10,978 protein-coding and 694 non-coding genes.

## Methods

### Sample acquisition and nucleic acid extraction

A male *Machimus atricapillus* specimen (idMacAtri3) was collected from Hartslock Reserve, Oxfordshire (latitude 51.511263, longitude –1.112222) on 20 August 2020, using an aerial net. The specimen was collected and identified by Sam Thomas (Natural History Museum) and preserved in liquid nitrogen. A second *M. atricapillus* specimen (idMacAtri1) was collected by Ryan Mitchell (Natural History Museum) from Hartslock Reserve, Oxfordshire (latitude 51.511263, longitude –1.112222) on 20 August 2020, using an aerial net. This specimen was used for RNA sequencing. A third *M. atricapillus* specimen (idMacAtri4) was collected by Liam Crowley (University of Oxford) from Wytham Woods, Oxfordshire (latitude 51.77, longitude –1.34) on 17 July 2021, using an aerial net. This specimen was also used for RNA sequencing.

DNA was extracted at the Tree of Life laboratory, Wellcome Sanger Institute (WSI). The idMacAtri3 sample was weighed and dissected on dry ice with tissue set aside for Hi-C sequencing. Abdomen tissue was disrupted using a Nippi Powermasher fitted with a BioMasher pestle. High molecular weight (HMW) DNA was extracted using the Qiagen MagAttract HMW DNA extraction kit. HMW DNA was sheared into an average fragment size of 12–20 kb in a Megaruptor 3 system with speed setting 30. Sheared DNA was purified by solid-phase reversible immobilisation using AMPure PB beads with a 1.8X ratio of beads to sample to remove the shorter fragments and concentrate the DNA sample. The concentration of the sheared and purified DNA was assessed using a Nanodrop spectrophotometer and Qubit Fluorometer and Qubit dsDNA High Sensitivity Assay kit. Fragment size distribution was evaluated by running the sample on the FemtoPulse system.

RNA was extracted from head and thorax tissue of idMacAtri1 and idMacAtri4 in the Tree of Life Laboratory at the WSI using TRIzol, according to the manufacturer’s instructions. RNA was then eluted in 50 μl RNAse-free water and its concentration assessed using a Nanodrop spectrophotometer and Qubit Fluorometer using the Qubit RNA Broad-Range (BR) Assay kit. Analysis of the integrity of the RNA was done using Agilent RNA 6000 Pico Kit and Eukaryotic Total RNA assay.

### Sequencing

Pacific Biosciences HiFi circular consensus and 10X Genomics read cloud DNA sequencing libraries were constructed according to the manufacturers’ instructions. Poly(A) RNA-Seq libraries were constructed using the NEB Ultra II RNA Library Prep kit. DNA and RNA sequencing were performed by the Scientific Operations core at the WSI on Pacific Biosciences SEQUEL II (HiFi) and Illumina NovaSeq 6000 (RNA-Seq) instruments. Hi-C data were also generated from head and thorax tissue of idMacAtri3 using the Arima v2 kit and sequenced on the Illumina NovaSeq 6000 instrument.

### Genome assembly

Assembly was carried out with Hifiasm ( Cheng *et al.*, 2021) and haplotypic duplication was identified and removed with purge_dups ( Guan *et al.*, 2020). One round of polishing was performed by aligning 10X Genomics read data to the assembly with Long Ranger ALIGN, calling variants with freebayes ( Garrison & Marth, 2012). The assembly was then scaffolded with Hi-C data ( Rao *et al.*, 2014) using YaHS ( Zhou *et al.,* 2023). The assembly was checked for contamination as described previously ( Howe *et al.*, 2021). Manual curation was performed using HiGlass ( Kerpedjisev *et al.*, 2018) and Pretext ( Harry, 2022). The mitochondrial genome was assembled using MitoHiFi ( Uliano-Silva *et al.*, 2022), which performed annotation using MitoFinder ( Allio *et al.*, 2020). The genome was analysed and BUSCO scores were generated within the BlobToolKit environment ( Challiss *et al.*, 2020). Table 3 contains a list of all software tool versions used, where appropriate.

**Table 3.** Software tools and versions used.

Software tool	Version	Source
BlobToolKit	3.4.0	Challis *et al.*, 2020
Hifiasm	0.16.1-r375	Cheng *et al.*, 2021
HiGlass	1.11.6	Kerpedjiev *et al.*, 2018
MitoHiFi	2	Uliano-Silva *et al.*, 2022
PretextView	0.2	Harry, 2022
purge_dups	1.2.3	Guan *et al.*, 2020
YaHS	yahs-1.1.91eebc2	Zhou *et al.*, 2023

### Genome annotation

The Ensembl gene annotation system ( Aken *et al.*, 2016) was used to generate annotation for the *M. atricapillus* assembly (GCA_933228815.1). Annotation was created primarily through alignment of transcriptomic data to the genome, with gap filling via protein to-genome alignments of a select set of proteins from UniProt ( UniProt Consortium, 2019).

### Ethics and compliance issues

The materials that have contributed to this genome note have been supplied by a Darwin Tree of Life Partner. The submission of materials by a Darwin Tree of Life Partner is subject to the Darwin Tree of Life Project Sampling Code of Practice. By agreeing with and signing up to the Sampling Code of Practice, the Darwin Tree of Life Partner agrees they will meet the legal and ethical requirements and standards set out within this document in respect of all samples acquired for, and supplied to, the Darwin Tree of Life Project. All efforts are undertaken to minimise the suffering of animals used for sequencing. Each transfer of samples is further undertaken according to a Research Collaboration Agreement or Material Transfer Agreement entered into by the Darwin Tree of Life Partner, Genome Research Limited (operating as the Wellcome Sanger Institute), and in some circumstances other Darwin Tree of Life collaborators.

## Data Availability

European Nucleotide Archive *Machimus atricapillus*. Accession number PRJEB50882; https://identifiers.org/ena.embl/PRJEB50882. ( Wellcomse Sanger Institute, 2022) The genome sequence is released openly for reuse. The *Machimus atricapillus* genome sequencing initiative is part of the Darwin Tree of Life (DToL) project. All raw sequence data and the assembly have been deposited in INSDC databases. Raw data and assembly accession identifiers are reported in Table 1.

## Author information

Members of the Natural History Museum Genome Acquisition Lab are listed here: https://doi.org/10.5281/zenodo.4790043.

Members of the University of Oxford and Wytham Woods Genome Acquisition Lab are listed here: https://doi.org/10.5281/zenodo.4789928.

Members of the Darwin Tree of Life Barcoding collective are listed here: https://doi.org/10.5281/zenodo.4893703.

Members of the Wellcome Sanger Institute Tree of Life programme are listed here: https://doi.org/10.5281/zenodo.4783585.

Members of Wellcome Sanger Institute Scientific Operations: DNA Pipelines collective are listed here: https://doi.org/10.5281/zenodo.4790455.

Members of the Tree of Life Core Informatics collective are listed here: https://doi.org/10.5281/zenodo.5013541.

Members of the Darwin Tree of Life Consortium are listed here: https://doi.org/10.5281/zenodo.4783558.
