# Supplementary material for: The genome sequence of the Kite-tailed Robberfly, Machimus atricapillus (Fallén, 1814)
Source: Wellcome Open Res. 2023 Mar 8;8:113. [Version 1] doi: 10.12688/wellcomeopenres.19080.1 (PMC10622858; doi:10.12688/wellcomeopenres.19080.1)
Supplement: Peer review history

# Reviewer report: Reviewer response for version 1

**DOI:** 10.21956/wellcomeopenres.21155.r67257

**Published:** 2023-11-16

**Authors:** Quentin Rougemont

**Affiliations:** 1 University of Montpellier, Montpellier, France

**Review of 'The genome sequence of the Kite-tailed Robberfly,** *Machimus atricapillus* **(Fallén, 1814)'**

 This is a high-quality chromosome level assembly of a new species the Kite-tailed Robberfly,

*Machimus atricapillus *that will benefit the whole community. More details may be provided on the scaffolding (e.g. The scaffolding is lacking details on the chromosome boundaries, and the middle part is poorly connected, so it is hard to assess the quality of the scaffolding of these areas. To which chromosome does it belong?). You could also elaborate on how X and Y were identified.

 Providing the busco score for the gene's annotations would be great. More details on the gene annotation pipeline would be useful. Moreover, repeated elements were certainly identified, and some quantitative numbers could be provided.

1. **Is the rationale for creating the dataset(s) clearly described? **Yes. It will be useful for ecology, evolution, and taxonomic questions.
2. **Are the protocols appropriate and is the work technically sound?** Yes.
3. **Are sufficient details of methods and materials provided to allow replication by others? **Yes. However, more details would be welcome regarding whether any custom parameters were used or only default parameters were used for all software (HiFiasm, YaHs, Long-Ranger, Freebayes, MitoHiFi, etc). Is the number of chromosomes identified in agreement with expectations from this genus?
4. **Are the datasets clearly presented in a usable and accessible format?** Yes.

Are sufficient details of methods and materials provided to allow replication by others?

Yes

Is the rationale for creating the dataset(s) clearly described?

Yes

Are the datasets clearly presented in a useable and accessible format?

Yes

Are the protocols appropriate and is the work technically sound?

Yes

Reviewer Expertise:

population genomics

I confirm that I have read this submission and believe that I have an appropriate level of expertise to confirm that it is of an acceptable scientific standard.

# Reviewer report: Reviewer response for version 1

**DOI:** 10.21956/wellcomeopenres.21155.r67245

**Published:** 2023-11-02

**Authors:** Kuppusamy Sivasankaran

**Affiliations:** 1 Division of Taxonomy and Biodiversity, Entomology Research Institute, Loyola College, Chennai, Tamil Nadu, India

I appreciate the authors for assembling the *Machimus atricapillus *(Fallén, 1814) **whole genome sequence. The authors have used the proper software for assembly and annotation of the genome. In genome assembly, the authors identified 10,978 protein-coding genes and 694 non-coding genes.

**Minor clarification:**

- Authors have not written the species name in italics in the abstract. It can be written in italics.
- Mitochondrial genome length 16, 324 bp not mentioned in the text. The mitogenome genome length can be included in the text.
- Page no 3: The last paragraph 5 ^th^ line “ *and the phylogeny of the wider*” was given in italics. The sentence can be changed to the regular font.
- Page no 5: The first paragraph starts, “The specimen was collected and identified…” The sentence can be modified as: “The collected specimen was identified by Sam Thomas (Natural History Museum) and preserved in liquid nitrogen."

 

 Overall, the manuscript can be accepted for indexing.

Are sufficient details of methods and materials provided to allow replication by others?

Yes

Is the rationale for creating the dataset(s) clearly described?

Yes

Are the datasets clearly presented in a useable and accessible format?

Yes

Are the protocols appropriate and is the work technically sound?

Yes

Reviewer Expertise:

Phylogenetic analysis of moths using mitogenomes

I confirm that I have read this submission and believe that I have an appropriate level of expertise to confirm that it is of an acceptable scientific standard.

# Reviewer report: Reviewer response for version 1

**DOI:** 10.21956/wellcomeopenres.21155.r64425

**Published:** 2023-11-02

**Authors:** Markus Friedrich

**Affiliations:** 1 Department of Biological Sciences, Wayne State University, Detroit, Michigan, USA

This report describes the sampling, sequencing, and assembly of the nuclear and mitochondrial genomes of the robberfly species *Machimus atricapillus*. All experimental procedures represent state-of-the-art protocols and bioinformatic tools and are described in adequate detail. This genome will represent a high-quality contribution to the Wellcome Trust Darwin Tree of Life project.

 Minor comment: "and the phylogeny of the wider" in the last paragraph of the Background section needs to be de-italicized.

Are sufficient details of methods and materials provided to allow replication by others?

Yes

Is the rationale for creating the dataset(s) clearly described?

Yes

Are the datasets clearly presented in a useable and accessible format?

Yes

Are the protocols appropriate and is the work technically sound?

Yes

Reviewer Expertise:

Gene family evolution, insects, visual system

I confirm that I have read this submission and believe that I have an appropriate level of expertise to confirm that it is of an acceptable scientific standard.

# Reviewer report: Reviewer response for version 1

**DOI:** 10.21956/wellcomeopenres.21155.r63078

**Published:** 2023-07-24

**Authors:** Alex Makunin

**Affiliations:** 1 Wellcome Sanger Institute, Hinxton, UK

The manuscript by Thomas et al. presents high-quality genome assembly for Kite-tailed Robberfly, *Machimus atricapillus*. The assembly results are presented in a concise yet detailed fashion. Gene annotation is presented as a very high level summary in both results and methods. 

 Small notes:

- Percentage of assembly sequence assigned to six chromosomal-level scaffolds (99.99%) does not seem to match Table 2 with 3.78Mbp unplaced scaffolds
- From Hi-C figure, it seems that chromosomes were oriented as short arm - centromere - long arm. If that is the case, can this be indicated in the genome report?
- Figure 5 would benefit from a grid separating chromosomes - at the moment it is unclear if the small block with small affinity with other genomic regions belongs to chrX or chr2. 

Are sufficient details of methods and materials provided to allow replication by others?

Yes

Is the rationale for creating the dataset(s) clearly described?

Yes

Are the datasets clearly presented in a useable and accessible format?

Yes

Are the protocols appropriate and is the work technically sound?

Yes

Reviewer Expertise:

comparative genomics

I confirm that I have read this submission and believe that I have an appropriate level of expertise to confirm that it is of an acceptable scientific standard.
